# Diagnostic Utility of Cytokeratin 17 Expression in Oral Squamous Cell Carcinoma: A Review

**DOI:** 10.7759/cureus.27041

**Published:** 2022-07-19

**Authors:** Ankita Gyanchandani, Samarth Shukla, Sunita Vagha, Sourya Acharaya, Ravindra P Kadu

**Affiliations:** 1 Pathology, Jawaharlal Nehru Medical College, Datta Meghe Institute of Medical Sciences, Wardha, IND; 2 Medicine, Jawaharlal Nehru Medical College, Datta Meghe Institute of Medical Sciences, Wardha, IND

**Keywords:** ck 17, broders grading, immunohistochemistry, histological grading, oral squamous cell carcinoma

## Abstract

One of the most common oral malignancies is oral squamous cell carcinoma (OSCC). Although the prevalence of oral cancer varies worldwide, it is generally agreed that the oral cavity is a common anatomical site for cancer, depending primarily on the country (and even particular region in some countries) and gender of the patients. Finding diagnostic markers for OSCC is critical for early diagnosis and personalised treatment of patients.

Because they are overexpressed in OSCC relative to normal mucosa, cytokeratins (CKs), intermediate filaments of the cytoskeletons, are possibilities for diagnostic markers of OSCC. CK17 should be targeted as a diagnostic marker for OSCC among the CKs, as multiple other CKs have been linked to the disease. This study aims to assess the immuno-histochemistry expression of CK17 and to investigate whether there is a link between CK17 and OSCC differentiation.

## Introduction and background

Oral cancer refers to a collection of tumours that can affect any part of the mouth, pharynx, or salivary glands. Oral squamous cell carcinoma (OSCC) is thought to account for more than 90% of all oral neoplasms [1]. Despite breakthroughs in therapeutic techniques, OSCC morbidity and mortality rates have remained relatively unchanged over the previous 30 years. Males have rates of 6.6/100,000 and 3.1/100,000 morbidity and mortality, respectively, whereas females have rates of 2.9/100,000 and 1.4/100,000. Oral cancer is also six times more likely to develop in alcohol drinkers than in non-drinkers [1]. The combination of tobacco and alcohol use constitutes a 15-fold risk of oral cancer for users compared to non-users [2]. Oral cancer has the highest prevalence among all cancers in India, according to recent studies [1,3]. The male-to-female incidence of oral cancer is 53,842, and the female-to-female incidence is 23,161. Changes in behavioural and lifestyle habits have been reported to affect men two to three times more than women in India [3]. Despite the ease with which the oral cavity can be examined for therapeutic purposes, OSCC is frequently discovered in its advanced stages. The most prevalent causes are an incorrect initial diagnosis and the patient's or attending physician's ignorance [3].

A person's genetic predisposition and exposure to environmental carcinogens such nicotine, alcohol, chemical carcinogens, UV or ionising radiation, and microorganisms interact to cause various molecular processes that lead to OSCC. Chronic exposure to carcinogens can harm both specific genes and bigger chunks of the genetic code, including chromosomes. Oncogenes that promote cell survival and proliferation may become mutated or amplified as a result of genetic damage [1].

Sadly, the majority of OSCC are discovered at a late stage of the disease, despite the fact that early OSCC has the best prognosis, particularly those that are well-differentiated and have not metastasized. The tumour, the treatment, and the patient are all important variables that can affect the prognosis of OSCC. However, the percentage of cases that survive for five years in the advanced stages is less than 12%. Within the first 30 months of their illness, most advanced OSCC patients often pass away. Finally, early diagnosis continues to be essential for OSCC therapy that is effective. Clinicians should be aware that red or white plaques, tumours, or isolated ulcers may be signs of certain conditions, especially if they persist for more than two weeks. A biopsy from the suspicious lesion is required in these situations [1]. Keratins (K) are a family of intermediate filament proteins found mostly in epithelial cells. They play a fundamental structural role in the formation of the cytoskeleton, which is critical for cell structure integrity and stability. CK17 should be targeted as a diagnostic marker for OSCC among the CKs, as multiple studies have shown that CK17 expression can be found in malignant tissues when compared to normal tissue [4].

## Review

Predisposing risk factors

The causes for OSCC comprise tobacco-associated intra-oral carcinogens, which may play a collaborative role in the development of oral carcinoma. It has been estimated that 75% of cases of oral carcinoma can be prevented by eliminating the consumption of alcohol and tobacco. For the rest 25% of patients who are unexposed to these factors, the causes of their tumours are not known. The disproportionately higher incidence of carcinoma of the head-neck in relation to other malignancies in India may be due to the use of tobacco in various forms, consumption of alcohol, low socioeconomic conditions related to poor hygiene, poor diet and rampant viral infections [2]. Although alcohol and tobacco use are commonly the main predisposing factors, there are also other causes such as betel nut chewing in certain culturally biased populations. Betel nut usage is common in the Asian subcontinental population and, therefore, is related to an increased risk of oral cancer. Other causes include narcotics [1].

In older males, low socioeconomic groups and certain culturally biased populations, OSCC is often observed. Other components that are involved are reduced ability to repair DNA damaged by mutagens, reduced ability to metabolize carcinogens, deficiencies of vitamins A, E or C or trace elements, and defects in the immune system. The inadequate immune response may predispose to cancer development. There is also an increased risk to develop OSCC in HIV-infected patients and patients subjected to organ transplantations and those who are under immunosuppressive therapy [1].

Clinical features of OSCC

This neoplasm can be seriously dangerous since, in the early stages, it may go undetected. The early stages are typically painless, but as it progresses, it could cause pain or a burning feeling. The tongue, lips, and mouth's floor are typical locations for OSCC to grow. Some OSCCs develop in the mucosa that appears to be normal, while others are preceded by premalignant lesions that are clinically visible, particularly erythroplakia and leukoplakia. Typically, OSCC manifests as an ulcer with elevated exophytic edges or fissuring. It can also appear as a lump, a red lesion (erythroplakia), a white or mixed white and red lesion, an extraction socket that won't heal, or an enlarged cervical lymph node that is hard or fixated. OSCC should be taken into account where any of these symptoms remain for more than two weeks [1].

Molecular pathogenesis of OSCC

The importance of inheritance in the development of oral cancer has been the subject of numerous investigations. First-degree relatives of oral cancer patients are at a higher relative risk of developing the disease, with odds ratios ranging from 1.1 to 3.8. Oral cancer is genetically predisposed, according to several genes. The increase in relative hazard among carriers is attributed to gene polymorphisms involved in the metabolism of xenobiotic substances, including glutathione S-transferase mu 1 (GSTM1) and cytochrome P450 1A1 (CYPIA 1). Oropharyngeal malignancies are more likely to develop in those with the genotype for alcohol dehydrogenase 3 [1].

Field cancerization is the theory of oral carcinogenesis. Theoretically, because the oral epithelium is exposed to carcinogenic agents, the region as a whole is at a higher risk of developing malignant lesions due to the buildup of hereditary oncogene and tumour suppressor gene changes. Multiple oral tumours may arise from separate cell clones, according to the cancerization field. Data from chromosomal X inactivation research, microsatellite analysis, and p53 mutational analysis have been used to support this idea [1].

Diagnosis of OSCC

OSCC can be diagnosed using a clinical examination and lymph node palpation. Vital staining (rose bengal, methylene blue, toluidine blue), histopathology, photospectrometry, fine needle aspiration cytology (FNAC), liquid-based cytology, and molecular analysis are some of the several diagnostic modalities. Histopathological examination is a pathologist's gold standard weapon when dealing with OSCC. [5] It's tough to tell what kind of tumour a poorly differentiated tumour is and how to tell them apart based on their morphology. As a result of this constraint, IHC and flow cytometry are widely used.

Histological grading for OSCC

TNM staging, Broder's system (1920), Jakobsons, Fisher (1975), Lund (1975), Willen (1975), Crissman, Anneroth, Bryne (1975), Crissman, Anneroth, Bryne (1975), Crissman, Anneroth, Bryne (1975), Crissman, Anneroth, Bryne (1975), Crissman, Anneroth, Bryne (1975), Cri (1989-92) are different grading systems used in OSCC [5]. In this investigation, we have used Broder's Histological Grading (Table 1).

**Table 1:** Broder's histological grading system

Grading of OSCC	Percentage of undifferentiated cells
Well differentiated (grade1) Undifferentiated cells	<25%
Moderately differentiated (grade 2) Undifferentiated cells	<50%
Poorly differentiated (grade 3) Undifferentiated cells	<75%
Anaplastic (grade 4) Undifferentiated cells	>75%

Immunohistochemistry for OSCC

IHC is a technique that uses antigen-antibody interactions to identify cellular or tissue components or antigens. In histology, it is employed as a diagnostic method. IHC can be used in everyday situations. It can be used with typical fixation and embedding techniques. It can be done in the past using archived material. It is sensitive and may be used with practically any immune molecule, and it's evaluated in terms of morphology [6]. Monoclonal and polyclonal antibodies are used in immunohistochemistry to assess the tissue distribution of an antigen in health and illness. The technique of IHC is commonly utilised in cancer diagnosis. IHC is used to identify a variety of proteins, enzymes, and tissue structures because it involves antigen-antibody reactions [7]. IHC markers have lately acquired popularity as a reliable diagnostic tool. Cancer is detected by IHC markers such as Ki-67, p53, CK17, CK13, laminin -52, and type IV collagen [8].

Cytokeratin 17

Cytokeratins are proteins that make up the epithelial cells' intermediate and main cytoskeletons. Low weight or basic or neutral type 2 cytokeratins are the two types of keratins. Cytokeratins of high molecular weight, also known as basic or neutral cytokeratins, include CK1, CK2, CK3, CK4, CK5, CK6, CK7, CK8, and CK9. On the other hand, CK10, CK12, CK13, CK14, CK16, CK17, CK18, CK19, and CK20 are low molecular weight cytokeratins or acidic cytokeratins. CK17 is a basal/myoepithelial cell-specific protein that can be induced in activated keratinocytes. K17 is absent in normal stratified epithelia, and its presence in comparable malignancies could be interpreted as neoexpression during carcinogenesis [9].

These cytokeratins express themselves differently in various organs, making them organ-specific. CK1 has the highest molecular weight, but CK19 has the lowest, as the molecular weight falls as the number increases. Depending on the kind of epithelium and the pattern of development, an epithelial cell expresses distinct subsets of cytokeratins. The structural integrity of the epithelium is safeguarded by keratins, which also control protein synthesis, signalling, growth, and motility. Historically, keratins have been utilised as diagnostic indicators. Nevertheless, mounting evidence supports their significance as prognostic indicators, active regulators of epithelial carcinogenesis, and predictors of response to therapy [9]. CK17 stained sections with <5% stained cells are considered to be negative and those with >5% stained cells are positive (Table 2).

**Table 2:** Methodology for Cytokeratin 17 expression

Percentage of stained cells	Intensity
5-30%	+
31-60%	++
>60%	+++

Matsuhira et al. carried out a study on CK13, CK17, ki 67, and p53 expression in the upper layers of epithelial dysplasia surrounding tongue squamous cell carcinoma. They concluded that strong expression of CK13 in normal epithelium, ki67 and p53 in lower layers of normal epithelium, CK13 and CK17 in upper layers of epithelial dysplasia and cancerous lesions, and CK17, Ki67, and p53 in lower layers of epithelial dysplasia and cancerous lesions were found in this study [10]. Sanguansin et al. conducted a study on CK17 expression in OSCC. It was concluded that CK17 expression was higher in OSCC than in normal oral mucosa [11]. CK17 and CK13 expression in oral epithelial dysplasia and OSCC were studied by Kiani et al. In this study, opposing expression of CK13 and CK17 was observed, with a decrease of CK13 and overexpression of CK17 associated with increased dysplasia and invasive cancer [12]. 

Wei et al. carried out a study on overexpression of CK17 protein in OSCC in vitro and in vivo. A total of six healthy persons and 30 primary OSCC patients were taken. The study revealed the increased CK17 expression in cancerous tissues from OSCC patients compared with paired adjacent non-malignant epithelia [13].

Nobusawa et al. conducted a study on immunohistochemical staining patterns of CK13, CK14, and CK17 in oral epithelial dysplasia including orthokeratotic dysplasia. It was found from the study that CK14 expression can be used to detect early epithelial dysplasia and that CK13 and CK17 expression are useful for detecting neoplastic changes [14]. Coelho et al. conducted research on keratin 17 and keratin 19 expression in OSCC. In this study, they analysed the expression of CK17 and CK19 in OSCC patients via IHC in tumoral and non-tumoral tissues. They concluded that CK17 and CK19 are highly expressed in OSCC in comparison to non-tumoral tissues. [15]. Kuijie et al. conducted a study on CK17 expression in OSCC. It was concluded that the increased CK17 gene may be associated with tumorigenesis and the development of OSCC [16]. 

Kiani et al. conducted a study on CK13 and CK17 expression in oral epithelial dysplasia and OSCC. Opposite expression of CK13 and CK17 was seen in this study, in the form of loss of CK13 and overexpression of CK17 with an increase in the degree of dysplasia and invasive carcinoma [17]. A number of different studies reported on OSCC [18-21]. Hande et al. reported on immunohistochemical analysis of tumour-associated stroma in OSCC [22]. Gadbail et. al reported four studies on the expression of Ki67, CD105, and α-SMA in OSCC [23-26]. Mohite et al. reported about the immunohistochemical evaluation of the expression patterns of P53, P63, and P73 in epithelial dysplasia [27].

In our study, it was observed that CK17 was strongly expressed in the majority of tumour cells in well-differentiated squamous cell carcinoma. CK17 is weakly expressed in the majority of tumour cells in moderately differentiated squamous cell carcinoma. CK17 was not expressed in a majority of tumour cells in poorly differentiated squamous cell carcinoma. The present study aims to close the gap of understanding between the expression of CK17 in OSCC. It will enable us to breach the gap in understanding the relationship between CK17 and histological grade in OSCC. It will help to predict the aggressiveness of the tumour, which helps in early diagnosis and treatment.

## Conclusions

OSCC is a common public health problem worldwide with a poor prognosis. Even though the oral cavity is easily accessible for a clinical examination, OSCC is typically only discovered in advanced stages. The most frequent causes are an incorrect initial diagnosis and ignorance on the part of either the patient or the treating physician. IHC markers have lately acquired popularity as a reliable diagnostic tool. CK17 expression in the detection of OSCC can be used as an adjunct in the grading of OSCC in correlation with Broders’ system. CK17 expression can be correlated with the Broders’ histological classification and, thus, it can be helpful in early diagnosis and also in determining the aggressiveness of the tumour more accurately so that we can decide the appropriate treatment.
